# Median Arcuate Ligament Syndrome in an Elderly Patient: A Symptomatic Post-stenotic Aneurysm and Systemic Atherothrombotic Disease

**DOI:** 10.7759/cureus.116095

**Published:** 2026-09-11

**Authors:** Leandro Gontijo, Pedro Enzo Camargo Luz, Matheus Cordón, Luiza Cordeiro, João Cordeiro, Eduardo Batista, Marina Penna, Halana Bezerra, Luiza Letti Ferronatto

**Affiliations:** 1 Surgery, Hospital de Base, Brasília, BRA; 2 Medicine, Centro Universitário de Brasília, Brasília, BRA; 3 Surgery, Hospital Universitário de Brasília, Brasília, BRA; 4 Medicine, Escola Superior de Ciências da Saúde, Brasília, BRA; 5 Medicine, Universidade Católica de Brasília, Brasília, BRA

**Keywords:** aneurysm, case report, dubar syndrome, median arcuate ligament syndrome, mesenteric ischemia

## Abstract

Median arcuate ligament syndrome (MALS) is atypical in elderly patients, in whom it frequently coexists with severe atherosclerotic disease. We report the case of a 77-year-old male with marked mitral regurgitation who presented with intestinal claudication and malnutrition. CT angiography demonstrated MALS associated with a fusiform aneurysm of the celiac trunk with dissection, along with 60% atherosclerotic stenosis of the superior mesenteric artery. Given the limiting ischemic presentation, the patient underwent laparoscopic release of the ligament. The procedure was complicated by avulsion of the celiac trunk and massive hemorrhage, requiring conversion to laparotomy and definitive ligation of the vessel. Postoperatively, the patient developed mixed shock and irreversible diffuse mesenteric ischemia, culminating in death. This case illustrates the high risk of hemorrhagic complications and the difficulty of surgical decision-making when mechanical compression overlaps with extensive degenerative vascular fragility in an elderly patient.

## Introduction

Median arcuate ligament syndrome (MALS), or Dunbar syndrome, results from extrinsic compression of the celiac trunk by the median arcuate ligament of the diaphragm [1-4]. Although historically attributed exclusively to vascular ischemia, current evidence highlights the predominance of a neurogenic component (resulting from chronic compression of the celiac plexus) in the genesis of abdominal pain and gastric motility disturbances [3,5-7].

The diagnosis is classically described in female patients between the fourth and sixth decades of life [3,8]. Presentation in elderly patients is uncommon and carries critical particularities. In this subgroup, MALS frequently coexists with systemic atherosclerotic disease, complicating the differentiation between extrinsic mechanical compression and intrinsic degenerative stenosis [3,9]. Furthermore, chronic hemodynamic stress (post-stenotic jet) can progress to major vascular complications, such as the formation of visceral aneurysms and dissections, dramatically increasing the risk of rupture and thromboembolic events [8,10-12].

This report describes the case of a 77-year-old patient diagnosed with MALS, presenting with intestinal claudication associated with a celiac trunk aneurysm with non-progressive dissection. The complexity of the presentation was compounded by significant atherosclerotic stenosis of the superior mesenteric artery and multiple cardiovascular comorbidities. The objective of this study is to discuss the diagnostic challenges and the high risks of surgical management in a setting of anatomical compression superimposed on a severe degenerative vascular burden in an elderly patient.

## Case presentation

A 77-year-old widowed male, a retired farmer and construction worker residing in a peri-urban area, with preserved family support and functional independence, presented with a medical history of dyslipidemia, glaucoma, and severe primary mitral valve regurgitation. His psychosocial and family history revealed former smoking (cessation more than 40 years prior), no alcohol use, and a negative family history for malignancy. Regarding prior interventions, he had a surgical history of cervical nodule resection several decades prior to presentation and had a chronically reduced right inguinal hernia.

The chief complaint was intestinal claudication manifested by insidious, colicky mid-abdominal pain over two months, associated with a 12 kg weight loss during that period and sitophobia. The patient sought care due to significant worsening of postprandial pain over the preceding 24 hours. On admission, physical examination showed he appeared in fair general condition and cachectic. The abdomen was soft, with a pulsatile, painless mass palpable deep in the mid-abdomen on palpation, without signs of peritonitis, and a hyperpulsatile right popliteal pulse.

The timeline of the current episode began at a lower-complexity secondary hospital, where an initial CT scan suggested a dissection flap of the celiac trunk. Due to the need for detailed vascular imaging and specialized intervention, the patient was transferred to our tertiary referral center. For further characterization, repeat CT angiography of the aorta and its branches was performed. This confirmed the definitive diagnosis of median arcuate ligament syndrome, demonstrating the compressed origin of the celiac trunk (Figure 1). This condition was associated with post-stenotic fusiform aneurysmal dilation (13 mm) and non-progressive dissection (Figure 2). Additionally, fibrocalcified atheromatous disease of the aorta and a plaque at the origin of the superior mesenteric artery (SMA) were identified, causing 50-60% stenosis. 

**Figure 1:**
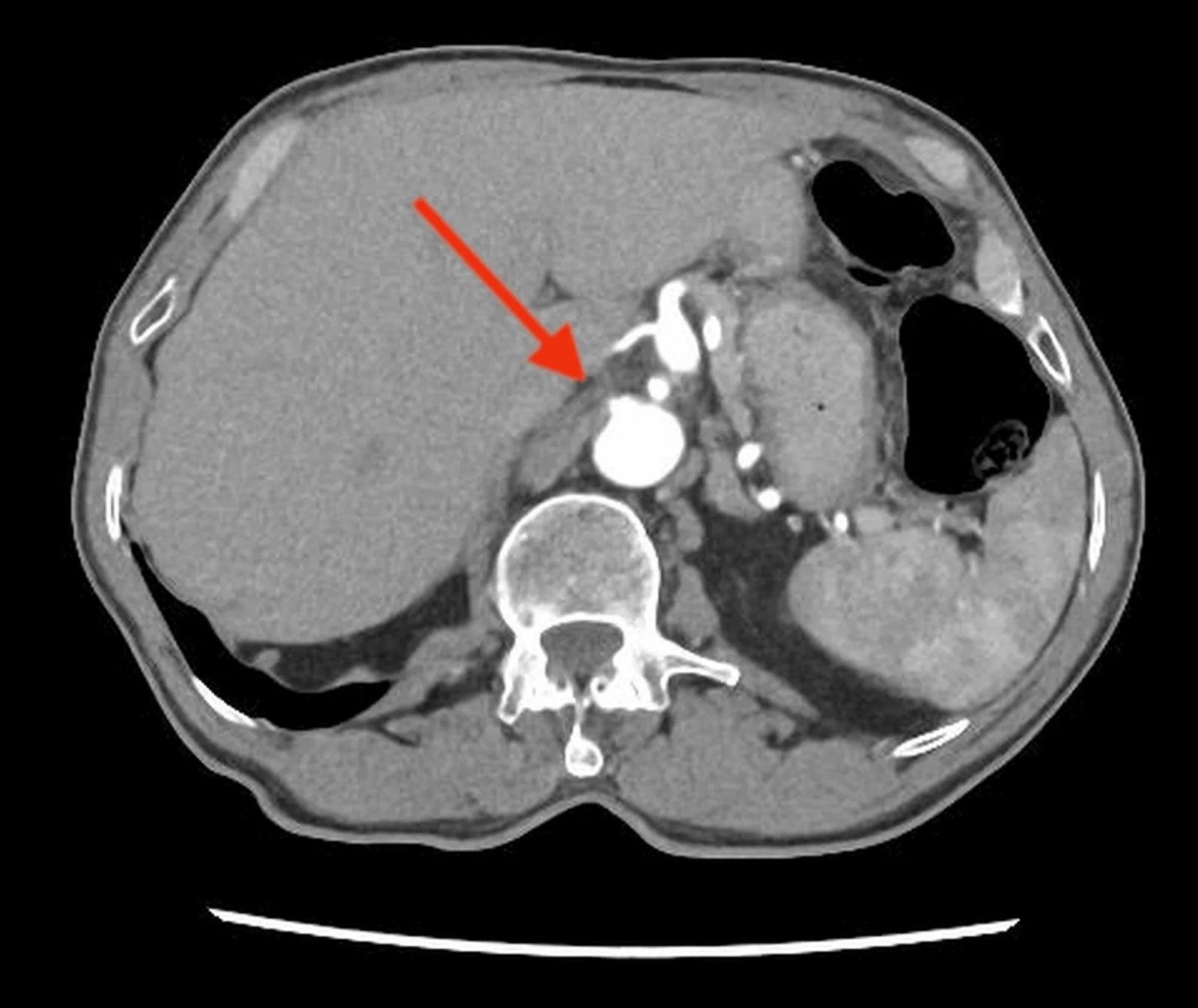
Contrast-enhanced axial computed tomography (CT) scan. The image demonstrates the exact origin of the celiac trunk from the abdominal aorta (arrow), highlighting its close proximity to the diaphragmatic crura, which form the median arcuate ligament responsible for the extrinsic compression.

**Figure 2:**
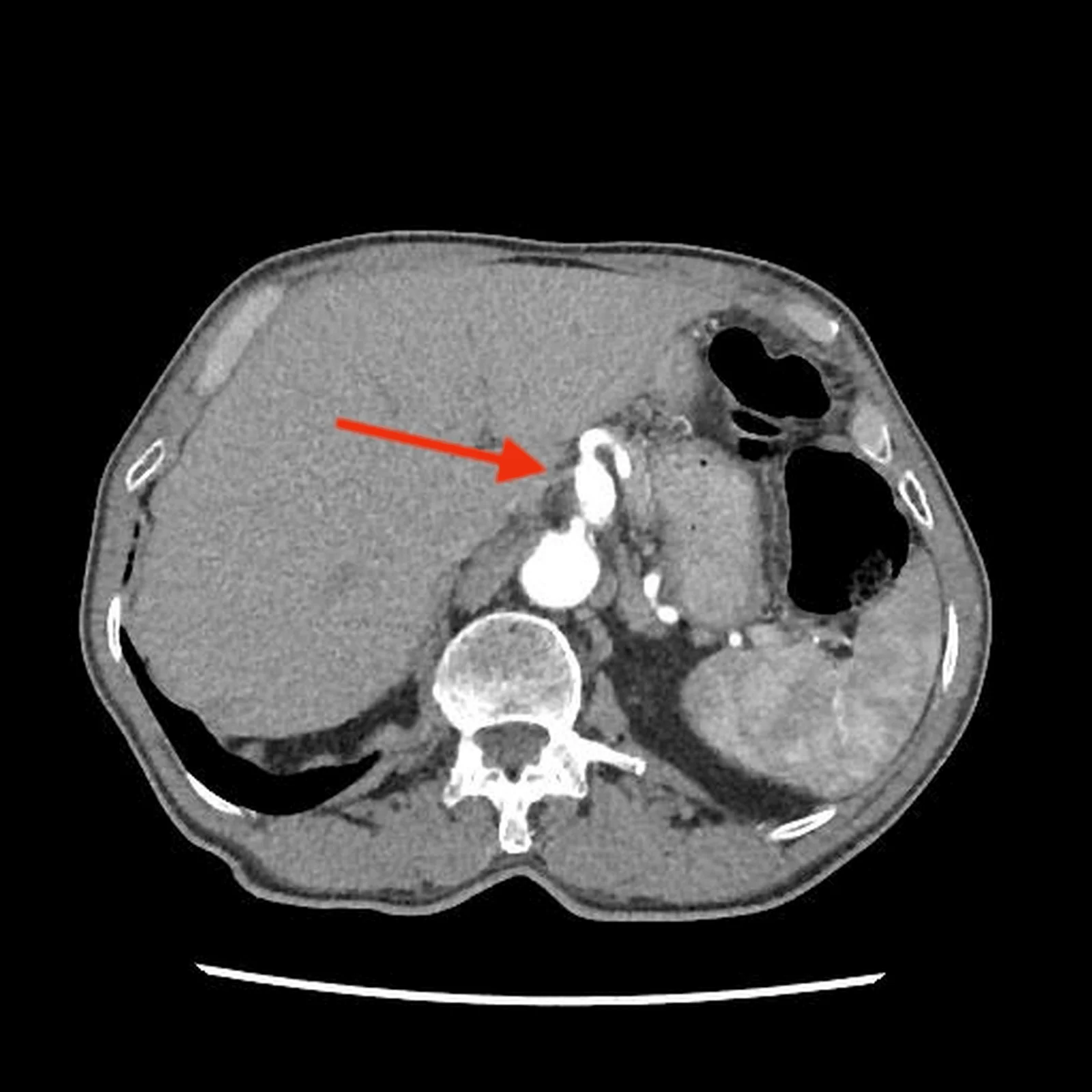
Contrast-enhanced axial computed tomography (CT) scan. A subsequent adjacent slice reveals the post-stenotic fusiform aneurysmal dilation of the celiac trunk (arrow), consistent with the hemodynamic repercussions of Dunbar syndrome.

During preoperative evaluation, it was noted that the patient had a prior diagnosis of severe primary mitral regurgitation (with transesophageal echocardiography showing extensive valve prolapse) and was being prepared for elective cardiac surgery. A multidisciplinary team evaluated the high risk of visceral aneurysm rupture and decided to postpone mitral valve replacement, prioritizing the immediate resolution of the abdominal pathology.

The initial therapeutic intervention (Day 1) consisted of laparoscopic release of the arcuate ligament, performed under antibiotic prophylaxis with a single preincisional intravenous dose of cefazolin 2 g. During intraoperative dissection, an unexpected and severe adverse event occurred: due to dense fibrotic adhesions between the degenerated aneurysmal wall and the ligament, mechanical traction resulted in complete avulsion of the celiac trunk and the right inferior phrenic artery, triggering massive hemorrhage. As local control proved impossible, emergency conversion to open laparotomy was warranted. Supraceliac aortic cross-clamping was performed for 25 minutes, with aortic repair using a bovine pericardial patch and definitive ligation of the avulsed vessel. Pharmacologic resuscitation required norepinephrine, tranexamic acid (1 g), and a massive transfusion protocol. Following the procedure, the patient was admitted to the intensive care unit in critical condition, with the initial postoperative laboratory findings summarized in Table 1. 

**Table 1:** Post-operative laboratory findings (Day 1) The table presents the patient's critical laboratory values obtained from the intensive care unit upon admission following the massive hemorrhagic event and emergency open laparotomy. All parameters reflect the acute hemodynamic and metabolic compromise resulting from the avulsion of the celiac trunk.

Parameter	Patient Result	Reference Range
Hemoglobin	12.1 g/dL	13.0-17.0 g/dL
Hematocrit	34.2%	39.0-50.0%
Leukocytes	16,680/mm³	4,000-10,000/mm³
Platelets	192,000/mm³	150,000-450,000/mm³
Creatinine	0.9 mg/dL	0.7-1.3 mg/dL
Urea	40 mg/dL	15-50 mg/dL
Potassium	4.92 mEq/L	3.5-5.1 mEq/L
Lactate (arterial gas)	68 mg/dL	<18 mg/dL
pH (arterial gas)	7.16	7.35-7.45

During follow-up in the intensive care unit, subsequent testing revealed refractory hyperlactatemia and intra-abdominal hypertension (IAP of 21 mmHg). A follow-up exploratory laparotomy (on Day 3) revealed diffuse mesenteric ischemia with extensive necrosis of the small bowel (from the ligament of Treitz onward) and generalized colonic compromise, along with splenic infarction and signs of hepatic hypoperfusion. Adherence to the care plan was complete, as it occurred in a setting of maximal organ support; however, the patient could not tolerate the intervention due to the splanchnic anoxic-ischemic insult. Given the anatomical infeasibility of digestive tract resection or reconstruction, the therapeutic approach was definitively changed from curative to palliative. Temporary abdominal closure was performed using a sterile 3-L polyvinyl chloride (PVC) intravenous fluid bag (custom-made Bogotá bag technique). The clinical outcome was multiple organ dysfunction, culminating in death by asystole on Day 4.

## Discussion

The management of this case presented significant clinical and diagnostic challenges. Initially, the diagnosis at the secondary hospital was limited to a suspected celiac trunk dissection, highlighting the diagnostic difficulty of MALS when patients are evaluated without specialized vascular imaging. Furthermore, a major therapeutic challenge emerged during the preoperative evaluation due to the patient's concurrent severe primary mitral regurgitation. Balancing the timing of elective cardiac surgery against the high-risk structural prognosis of a visceral aneurysm required a careful multidisciplinary approach. The decision to prioritize the abdominal pathology was driven by the imminent and fatal risk of aneurysm rupture.

This case report aims to describe the clinical, tomographic, and surgical progression of a lethal and atypical complication of MALS, illustrating the catastrophic consequences of mechanical compression overlapping with atherosclerotic disease. We acknowledge inherent limitations in our report, notably the absence of early external imaging and intraoperative photographs due to the extreme nature of the emergency. Furthermore, the impossibility of testing endovascular rescue strategies during the acute intraoperative shock restricted our evaluation solely to the outcome of the emergency open approach.

In the literature, MALS is predominantly diagnosed in young women, typically between the third and fifth decades of life (20-40 years), often thin and without cardiovascular risk markers [2,8]. Symptom onset in an elderly patient (77 years) deviates from this classic profile. In this subgroup, coexisting systemic atherosclerotic disease confounds the diagnosis, making it difficult to distinguish pure extrinsic mechanical compression from intrinsic arterial degeneration.

The rationale for the development of the celiac aneurysm and dissection is based on sustained hemodynamic stress. The literature indicates that chronic compression by the arcuate ligament generates prolonged turbulent flow (post-stenotic jet), which damages the endothelium and promotes severe structural degeneration, culminating in the formation of visceral aneurysms and wall dissections [8,10]. This alteration renders the vessel fragile and prone to intraoperative rupture.

From a surgical standpoint, the laparoscopic approach has become established as the primary gold standard due to lower morbidity and rapid relief of the neurologic and vascular symptoms associated with compression. However, this access carries critical disadvantages, notably the risk of iatrogenic injury to structures adjacent to the aorta. Retrospective studies show that 9.1% of laparoscopic approaches for MALS require emergency conversion to open surgery specifically due to massive bleeding, involving injuries to the celiac trunk itself, aortic puncture, or laceration of the phrenic artery [13-15]. In the present case, dense surrounding fibrosis and aneurysmal fragility precipitated complete avulsion of the trunk and the phrenic artery, dramatically reproducing the worst reported statistics for this procedure.

The pathophysiological rationale for the fatal ischemic outcome lies in the failure of the collateral network in the face of an absolute ischemic insult. Occlusion or ligation of the celiac trunk is commonly compensated for by retrograde flow from the superior mesenteric artery through the pancreaticoduodenal arcades [3,10,16]. However, due to severe systemic atherothrombotic disease, the patient had obstructive stenosis (50-60%) at the origin of the superior mesenteric artery. The limitation of this hemodynamic rescue pathway, combined with profound hemorrhagic shock and the 25 minutes of supraceliac aortic cross-clamping required to control the avulsion, led to an unsustainable splanchnic anoxic-ischemic insult. The result was refractory hyperlactatemia and diffuse ischemia of the small bowel and colon, precluding resection or reconstruction.

## Conclusions

In conclusion, the management of MALS combined with visceral aneurysms and atherosclerotic stenosis in elderly patients represents a scenario of extremely high complexity. This case demonstrates that, despite the benefits of laparoscopy, manipulation of structurally degenerated arteries carries a lethal risk of avulsion and hemorrhage. The main lesson from this report is the imperative need to assess the functional reserve of the mesenteric network; in the presence of limiting stenoses in the collateral flow and tissue fragility, primary open surgical approaches or preplanned hybrid strategies should be rigorously considered to avoid irreversible ischemic catastrophe.
